# Novel insect-specific flavivirus isolated from northern Europe

**DOI:** 10.1016/j.virol.2012.08.038

**Published:** 2012-11-25

**Authors:** Eili Huhtamo, Gregory Moureau, Shelley Cook, Ora Julkunen, Niina Putkuri, Satu Kurkela, Nathalie Y. Uzcátegui, Ralph E. Harbach, Ernest A. Gould, Olli Vapalahti, Xavier de Lamballerie

**Affiliations:** a Infection Biology Research Program, Research Programs Unit, Department of Virology, Haartman Institute, Faculty of Medicine, PO Box 21 (Haartmaninkatu 3), University of Helsinki, Helsinki FIN-00014, Finland; b Unité des Virus Emergents UMR190 “Emergence des Pathologies Virales”, Aix-Marseille Univ., Institut de Recherche pour le Développement, EHESP French School of Public Health, Marseille, France; c Natural History Museum, Cromwell Road, London, SW7 5BD, UK; d Department of Virology and Immunology, Helsinki University Central Hospital Laboratory, PO Box 400 (Haartmaninkatu 3), 00029 HUS, Finland; e Division of Microbiology and Epidemiology, Department of Basic Veterinary Sciences, PO Box 66 (Agnes Sjöbergin katu 2), University of Helsinki, Helsinki FIN-00014, Finland

**Keywords:** Mosquito, Flavivirus, Insect-specific flavivirus

## Abstract

Mosquitoes collected in Finland were screened for flaviviral RNA leading to the discovery and isolation of a novel flavivirus designated Hanko virus (HANKV). Virus characterization, including phylogenetic analysis of the complete coding sequence, confirmed HANKV as a member of the “insect-specific” flavivirus (ISF) group. HANKV is the first member of this group isolated from northern Europe, and therefore the first northern European ISF for which the complete coding sequence has been determined. HANKV was not transcribed as DNA in mosquito cell culture, which appears atypical for an ISF. HANKV shared highest sequence homology with the partial NS5 sequence available for the recently discovered Spanish *Ochlerotatus flavivirus* (SOcFV). Retrospective analysis of mitochondrial sequences from the virus-positive mosquito pool suggested an *Ochlerotatus* mosquito species as the most likely host for HANKV. HANKV and SOcFV may therefore represent a novel group of *Ochlerotatus*-hosted insect-specific flaviviruses in Europe and further afield.

## Introduction

Flaviviruses (family *Flaviviridae*, genus *Flavivirus*) are enveloped, positive sense single-stranded RNA viruses that include several important human pathogens such as dengue, yellow fever and Japanese encephalitis viruses. The flavivirus genome contains one open reading frame (ORF) that encodes a large polyprotein.This is processed by viral and host proteases to form the structural proteins C, preM and E, in addition to the seven non-structural proteins (NS1, NS2A, NS2B, NS3, NS4A, NS4B and NS5) (Chambers et al., 1990). Most of the known flaviviruses are vector-borne, being transmitted via mosquitoes or ticks between vertebrate hosts. However, some flaviviruses appear to be independent of vector transmission (Porterfield, 1980, Kuno et al., 1998). More recently, a large group of flaviviruses that apparently do not have vertebrate hosts, so-called “insect-specific” flaviviruses (“ISFs”), have also been identified (Cook and Holmes, 2006, Cook et al., 2006, Cook et al., 2011, Kuno, 2007). Since the discovery of cell fusing agent virus (CFAV) from a mosquito cell line (Stollar and Thomas, 1975, Cammisa-Parks et al., 1992), several related ISFs have been isolated and identified in field-collected mosquitoes from various geographic locations, including Africa (Kenya, Uganda) (Cook et al., 2009, Crabtree et al., 2003, Sang et al., 2003), Asia (Vietnam) (Crabtree et al., 2009), the Caribbean (Puerto Rico, Trinidad) (Cook et al., 2006, Kim et al., 2009), Central America (Guatemala) (Morales-Betoulle et al., 2008), North America (Mexico, Canada, USA) (Blitvich et al., 2009, Bolling et al., 2011, Farfan-Ale et al., 2009, Kim et al., 2009, Tyler et al., 2011). Using molecular methods, others have been detected but not isolated. These are reported from Europe (Italy, Spain, Portugal, Czech Republic, United Kingdom) (Aranda et al., 2009, Calzolari et al., 2010, Calzolari et al., 2012, Roiz et al., 2009, Sanchez-Seco et al., 2010, Vázquez et al., 2012) and Japan (Hoshino et al., 2007, Hoshino et al., 2009). The ISFs possess potentially distinct and unique features among flaviviruses. They have been shown to produce DNA forms of their genomic RNA (Crochu et al., 2004, Cook et al., 2009). In addition, integrated sequences related to insect-specific flaviviruses are present in the genomes of *Stegomyia aegypti* (=*Aedes aegypti*) and *Stegomyia albopicta* (=*Aedes albopictus*) mosquitoes (Crochu et al., 2004, Vázquez et al., 2012) and possibly other mosquitoes (nomenclature for the aedine mosquitoes follows Reinert et al., 2009). Therefore discrimination between DNA or RNA is essential for the detection of ISF sequences, and virus isolation in cell culture is the only robust demonstration of the presence of replicating virus. Another essential difference between the ISFs and other flaviviruses is that the ISFs encode an additional protein as an overlapping gene within the NS2A/NS2B coding sequence, a protein that may be produced via ribosomal frameshifting (Firth et al., 2010).

The ISFs isolated to date have been found mainly in mosquitoes but related sequences have been detected in phlebotomine sandflies (Moureau et al., 2010, Sanchez-Seco et al., 2010). The ISFs represent a phylogenetically distinct lineage in the genus *Flavivirus* and can be roughly grouped according to their host mosquito species (Hoshino et al., 2009). There is no statistical support for virus-mosquito co-divergence based on data currently available for the ISFs, suggesting that the ISFs may have undergone multiple introductions with frequent host-switching (Cook et al., 2011). ISFs or sequences related to them have been increasingly reported from mosquitoes collected in various geographical locations, including southern Europe. This has raised the issue of their potential presence in northern Europe. Previously, mosquitoes collected in Finland were studied by virus isolation and viral antigen screening, leading to the discovery of Lammi virus (LAMV), a novel flavivirus related to the classical mosquito-borne flaviviruses (Huhtamo et al., 2009). In this study, mosquitoes collected in Finland were screened for the first time by universal flavivirus RT-PCR. Using this method, a novel ISF was detected, subsequently isolated and fully characterized.

## Results

### Virus isolation and characterization

**Fig. 1:**
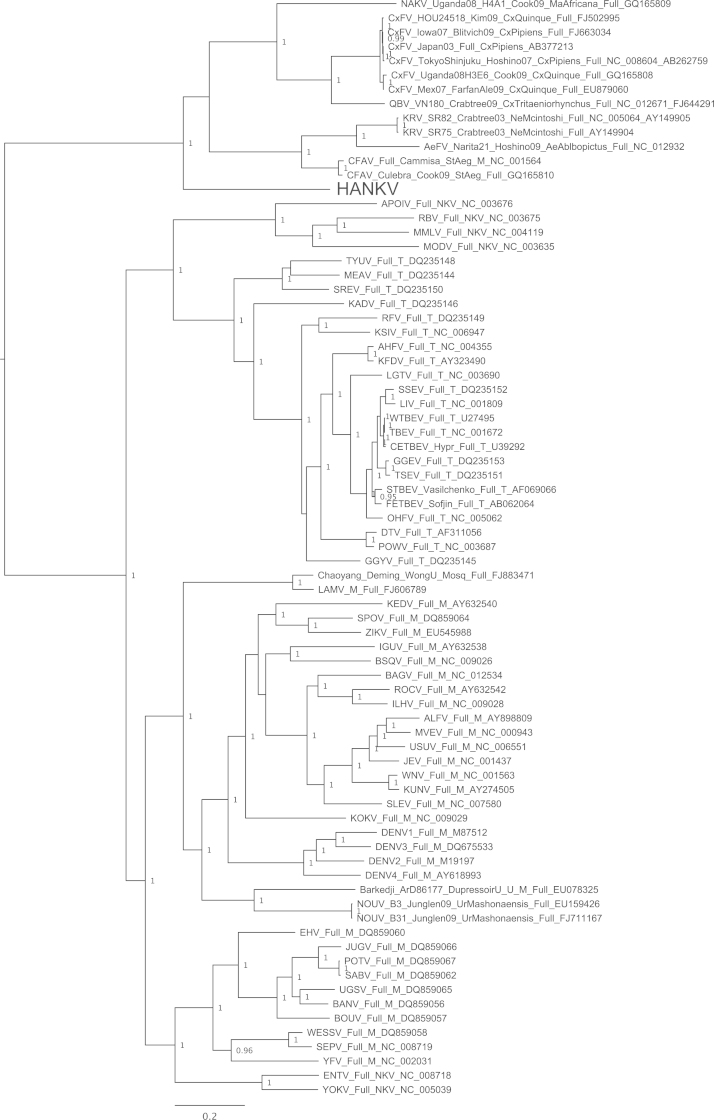
Bayesian phylogeny of the complete ORF amino acid data set with GBlocks stripping of regions of ambiguous alignment. Posterior probabilities of ≥0.9 for major nodes only are included and the tree is mid-point rooted for clarity. All horizontal branch lengths are drawn to a scale of substitutions per site. HANKV: Hanko virus. The rest of the virus abbreviations are according to ICTV 2011 (Pletnev et al., 2011).

**Fig. 2:**
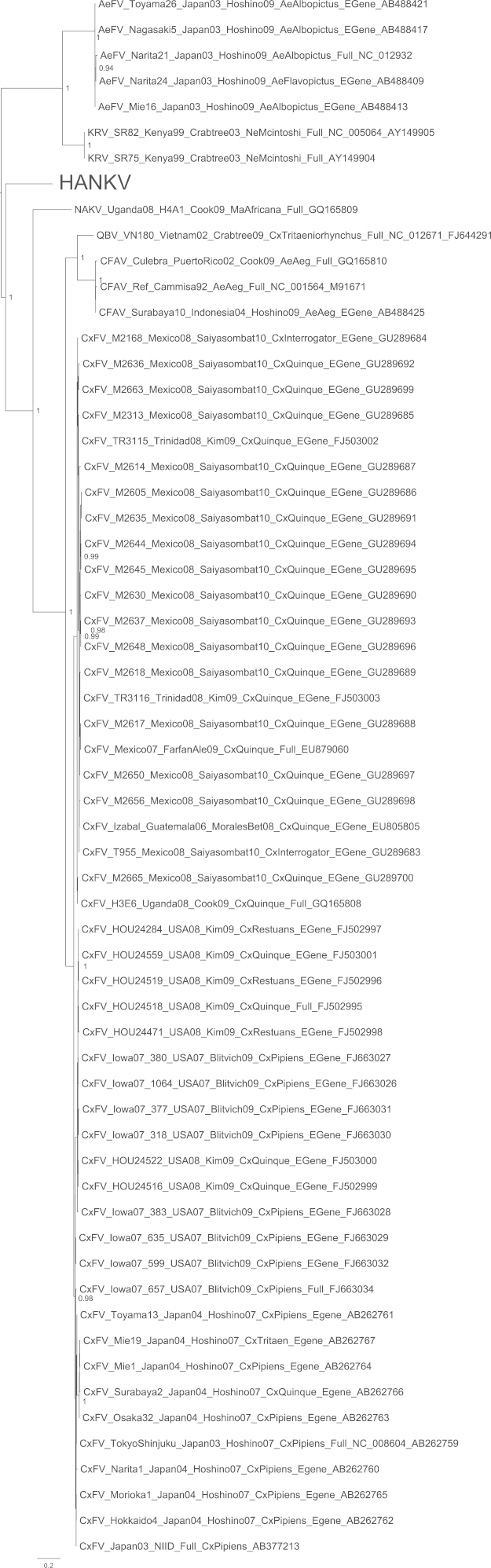
Bayesian phylogeny of the encoded E gene region amino acid data set. Posterior probabilities of ≥0.9 for major nodes only are included and trees are mid-point rooted for clarity. All horizontal branch lengths are drawn to a scale of substitutions per site. HANKV: Hanko virus. The rest of the virus abbreviations are according to ICTV 2011 (Pletnev et al., 2011).

**Fig. 3:**
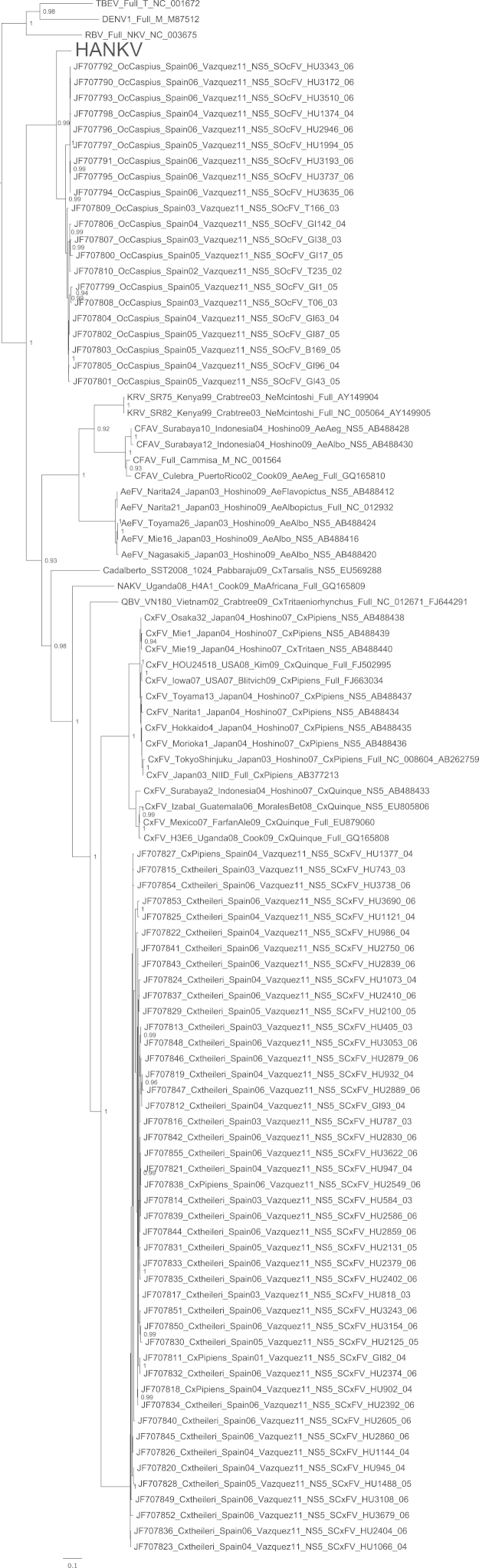
Bayesian phylogeny of the encoded NS5 gene region nucleotide data set including SOcFV isolates. Posterior probabilities of ≥0.9 for major nodes only are included and trees are mid-point rooted for clarity. All horizontal branch lengths are drawn to a scale of substitutions per site. HANKV: Hanko virus, SCxFV: Spanish Culex flavivirus, SOcFV: Spanish Ochlerotatus flavivirus (Vazquez et al., 2012). The rest of the virus abbreviations are according to ICTV 2011 (Pletnev et al., 2011).

A single mosquito pool was found positive via universal flaviviral RT-PCR screening and subsequently a virus was isolated in C6/36 cells. The isolate was named Hanko virus (HANKV) reflecting the name of the collection site of the mosquitoes. HANKV caused very mild CPE in C6/36 cells on day 7 post-infection that appeared as loosely attached and rounded cells. Cell fusion similar to that reported for CFAV in the same cell line (Stollar and Thomas, 1975) was not observed. In addition, the viral isolate did not cause CPE or produce detectable amounts of viral RNA in experimentally infected Vero E6 cells. Lack of cell fusion was also confirmed at low pH (5 and 6) in the presence of actively replicating virus. When examined by electron microscopy, concentrated HANKV virions appeared flavivirus-like and spherical, approximately 40–50 nm in diameter. The ORF sequence of HANKV was found to be 10,158 nucleotides long, encoding a polyprotein of 3385 amino acids (GenBank accession JQ268258). In phylogenetic analyses, HANKV grouped most closely with the ISFs. Nevertheless, the HANKV ORF sequence shared only 43–48% nucleotide and 40–42% amino acid identities with the ISFs, Cell Fusing Agent virus (CFAV), Aedes flavivirus (AeFV), Culex flavivirus (CxFV), Kamiti River virus (KRV), Quang Binh virus (QBV) and Nakiwogo virus (NAKV). In phylogenetic analyses of the complete ORF (Fig. 1), or the region encoding the NS3 gene (Supplementary Fig.), HANKV was positioned basal to the ISF group and was not significantly more closely related to the viruses associated with mosquitoes of the tribe Aedini (CFAV+AeFv+KRV), the genus *Culex* (CxFv, QBV) or the separate lineage of Nakiwogo virus (NAKV) isolated from a species of genus *Mansonia*. On the other hand, in the midpoint-rooted analysis of the E gene region, which did not include dengue, Rio Bravo or tick-borne encephalitis virus outgroups, HANKV appeared to fall in a different position (Fig. 2), and was more closely related to the Aedini-associated viruses, rather than the Culicini-related viruses. In an additional analysis including partial NS5 sequences of novel ISF-like sequences recently identified by Vazquez et al. (2012), HANKV grouped with Spanish Ochlerotatus flavivirus (SOcFV) sequences obtained from *Ochlerotatus caspius* from Spain. Within the available 917 bp NS5 sequence, HANKV and SOcFV sequences shared approximately 87–88% nucleotide and 97–99% amino acid identities. For comparison, within this region HANKV and other viruses previously isolated from mosquitoes of the tribe Aedini (CFAV, KRV, CxFv, AeFv, QBV and NAKV) shared 61.8–63.9% nucleotide and 64–68% amino acid homologies. HANKV and SOcFV isolates were found to group closely together and formed an outgroup to the rest of the tribe Aedini (Fig. 3). A preliminary analysis was also conducted incorporating ISF-related sequences recently collated from a number of different areas within Europe (Calzolari et al., 2012) which indicated that HANKV groups with a range of very short partial flavivirus-like sequences obtained primarily from *Ochlerotatus* mosquito species from Spain, Italy and Portugal. Since sequences were only ∼150 bp in length (NS5 gene region), and for most PCR positive pools in that respective study virus isolation was attempted but not successful, it is not clear if these sequences represent DNA integrations rather than virus. Thus, data are not shown for this additional analysis.

The HANKV genome included regions of sequence that appeared similar to those associated with ribosomal frameshift and additional protein production in other ISFs (Firth et al., 2010). No DNA forms of the HANKV genome were found using HANKV-specific primers in direct PCR, whereas the RT-PCR showed the presence of RNA using the same primer pairs. Control PCRs targeted to Cell silent agent (CSA) integrated DNA sequences CSA 1, CSA 2 and CSA 3 in the *St. albopicta* genome (Crochu et al., 2004), were positive from uninfected C6/36 nucleic acid extracts, further supporting the validity of the HANKV-negative DNA results. Immunofluorescence assays of HANKV-infected cells using hyperimmune antisera and MAbs raised against defined flaviviruses were also negative, supporting the evidence that HANKV is antigenically very distinct from the currently recognized flaviviruses.

### Vector mosquito identification

**Fig. 4:**
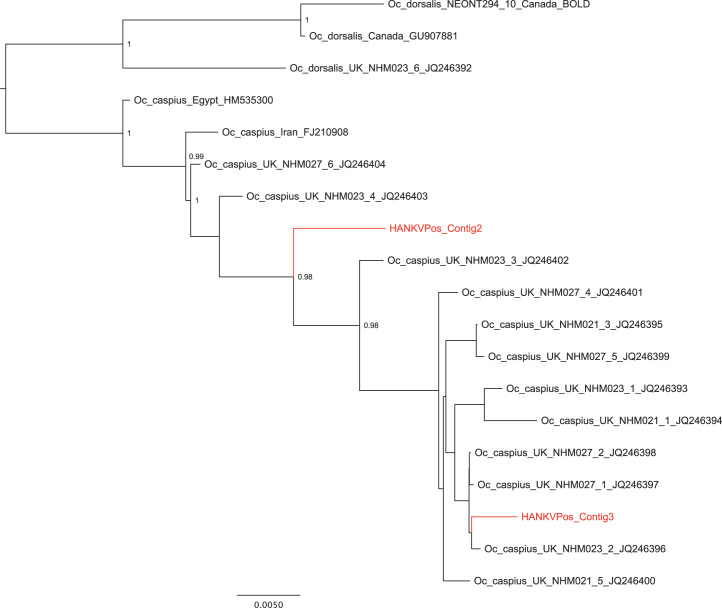
Bayesian phylogeny of *Oc. caspius* subset alignment for partial COI region, including *Oc.caspius* and *Oc.dorsalis* from a range of locations and clones Contig 2 and Contig 3 from HANKV-positive pools. For clarity, posterior probabilities obtained via Bayesian methods are shown for the main clades only. All horizontal branch lengths are drawn to scale. The tree is rooted on *Oc. dorsalis* for purposes of clarity only. Accession numbers and locations for sequences used are shown in the figure.

Retrospective identification of the mosquito species present in the HANKV-positive pool was attempted using amplified and cloned cytochrome C oxidase gene (COI) sequences obtained from the mosquito DNA present in the pools (designated Contigs 1, 2 and 3, respectively). Results from Bayesian phylogenetic analyses suggested that there were 2 potential host species for HANKV. The obtained COI sequences grouped with either *Ochlerotatus punctor* (Contig 1, not shown) and/or *Oc. caspius* (Contigs 2 and 3), with high levels of support as assessed using posterior probability values. Both of these species have been identified in Finland (Utrio, 1979). Considering the fact that within the currently available NS5 region, HANKV and SOcFV strains share approximately 99% amino acid identities and the latter virus was isolated from *Oc. caspius* from Spain, it appears most likely that the “host” mosquito species for HANKV is *Oc. caspius*. For clarity, phylogenetic analyses are presented using a data subset containing Contig 2, Contig 3 and *Oc. caspius* and *Oc. dorsalis* sequences from a range of locations worldwide. Contig 2 and Contig 3 group within the *Oc. caspius* clade with high levels of support, as assessed using posterior probability values (Fig. 4).

## Discussion

Previously, Lammi virus was isolated from mosquitoes in Finland (LAMV) (Huhtamo et al., 2009) and was phylogenetically closely related to the ‘classical’ mosquito-borne flaviviruses. In this study, a novel flavivirus was detected from Finnish mosquitoes by flaviviral RNA screening, designated as Hanko virus (HANKV), that was more closely related to the newly described group of ISFs and in particular the recently identified Spanish SOcFV isolated from *Oc. caspius*. Whereas LAMV was antigenically cross-reactive with other flaviviruses, the same flavivirus antibodies failed to react with HANKV antigens. The results of the phylogenetic analysis suggested that HANKV was distantly related to the other ‘classical’ members of the family which could explain the lack of serological cross-reactivity between them. Currently, the data on antigenic properties of ISFs is scarce; however, polyclonal hyperimmune antisera raised against mosquito-borne JEV and SLEV have been reported to be reactive with insect-specific CxFV in an IFA (Kim et al., 2009). The antigenic properties of the ISFs clearly merit further study, particularly in view of the fact that they are one defined criteria for flavivirus species differentiation (Pletnev et al., 2011)

HANKV shares several properties with the other ISFs. Its genomic sequence is more closely related to them than it is to the arthropod-borne flaviviruses and it was not found to infect vertebrate cells. Also in common with the ISFs, HANKV possesses a potential additional reading frame that presumably encodes a frameshift protein. In contrast, the lack of a DNA form appears to be a rare trait compared with the previously characterized ISFs (Cook and Holmes, 2006, Cook et al., 2009, Crochu et al., 2004) since the ability to produce DNA forms of the viral genome was previously believed to be a specific property shared by ISFs that could distinguish them from “classical” flaviviruses. However, very recently a novel ISF was detected in Portugal, Culex theileri flavivirus, for which no DNA forms were found (Parreira et al., 2012).

HANKV did not induce a cell fusion-type of CPE or syncytium formation in infected mosquito cells, which has been reported for some other ISFs (Cook et al., 2009, Kim et al., 2009, Stollar and Thomas, 1975). These properties are currently not defined for the other reported European ISF-like sequences and/or insect-specific flaviviruses (Calzolari et al., 2012, Vázquez et al., 2012). However, these results strongly suggest that the ISF lineage includes several groups of viruses with markedly different replication properties that may be associated with different hosts and their characteristics.

The independent results of phylogenetic analyses of complete ORF, NS3 and NS5 genes each define a similar phylogenetic position for HANKV, appearing outside the main cluster of *Stegomyia-* and *Culex*-associated ISFs. However, in the E gene analysis, HANKV was positioned inside the “insect-specific” group. This result most likely reflects a lack of available data for European *Ochlerotatus*-associated flaviviruses, since only partial NS5 sequences could be compared with HANKV and its most homologous virus, SOcFv. Alternatively, the results may indicate a recombinant history for the virus, similar to that suggested for CFAV (Cook et al., 2011). Clearly, the currently available partial NS5 genes of SOcFV and the short European ISF-like and/or ISF sequences (Calzolari et al., 2012, Vázquez et al., 2012) are inadequate for in depth phylogenetic and phylodynamic analyses of European ISFs. Very recently, the complete genome sequence was determined for C. theileri flavivirus (Parreira et al., 2012), which was shown to be genetically closely related to CxFv and QBV, but not to SOcFv. It may be that C. theileri flavivirus represents a distinct ISF lineage from HANKV, although the *C. theileri* flavivirus sequence was not included in our analysis as it was not available in the public databases.

In common with the Spanish SOcFV isolates, the mosquito mitochondrial sequences cloned from the isolated mosquito pool suggest that HANKV was probably present in an *Ochlerotatus* species, most likely *Oc. caspius*. However, it should be noted that this COI-based identification may be biased due to the lack of comparable sequence data for some mosquito species recorded in Finland, and also affected by the PCR amplification and cloning efficiencies. It is clear that viral surveillance studies in future should involve individual collection and homogenization of mosquitoes in preference to pooling and development of integrated morphological and molecular identification protocols.

Many of the recently reported ISFs from Europe are based on very short NS5 gene sequences amplified directly from mosquitoes (collated by Calzolari et al., 2012). Without further evidence they cannot be distinguished from possible ISF-like DNA integrations in mosquito genomes. This is even more likely in view of the fact that the majority of viral isolation attempts to date have been unsuccessful (Calzolari et al., 2012). To the best of our knowledge, HANKV is the first member of the ISF group to be isolated and characterised from northern Europe. Based on the current information, HANKV has several distinct features compared with the previously characterized ISFs and together with SOcFV, may represent a novel *Ochlerotatus*-hosted group of ISFs in Europe.

## Materials and methods

### Flavivirus screening of mosquitoes

The field specimens examined in this study included 450 mosquitoes collected in August 2005 at Tvärminne Zoological Station in Hanko peninsula, located on the southern coast of Finland (59° 50′ N, 23° 15′ E). Mosquitoes were collected using hand nets and placed in 1.5 ml Eppendorf tubes, 10 mosquitoes per tube and stored at −70 °C prior to use. Mosquitoes were homogenized with sterilized sand into 400 μl of Dulbecco's PBS+0.2% BSA and antibiotics, RNA was extracted using Qia Amp viral RNA mini kit (Qiagen) from each140 μl of mosquito homogenate. RT-PCR screening of material was conducted using flavivirus-specific primers CFD2 and MAMD targeted to a conserved region of the flavivirus NS5 gene (Scaramozzino et al., 2001). Reverse transcription reactions were performed using Expand reverse transcriptase (Roche) and PCR using Recombinant *Taq* DNA polymerase (Fermentas).

### Virus isolation and identification

Virus isolation was attempted from mosquito homogenates after passage through a 0.45 nm filter onto subconfluent C6/36 *Stegomyia albopicta* (=*Aedes albopictus*) cells. The monolayers were observed for cytopathic effects (CPE) and harvested when 50% of the monolayer was affected. An aliquot of infectious supernatant medium was used to infect fresh C6/36 cells, from which supernatants were harvested and virions concentrated using Millipore 100 kDa cut off columns. Recovered virus was examined by electron microscopy and also used for genome amplification. Electron microscopy was performed on concentrated virions on copper grids after negative staining with 2% KPTA (tungstophosphoric acid). Mammalian Vero E6 cells were also infected with supernatant medium harvested from infected C6/36 cells and observed for 14 day prior to harvesting cells and supernatants for analysis.

HANKV-infected C6/36 cells were examined in an immunofluorescence assay (IFA) using flavivirus envelope-specific MAbs 813 (Gould et al., 1985) and HB-112 (Henchal et al., 1982) and a known flavivirus IgG-positive human serum. For the detection of possible DNA forms of HANKV, both DNA and RNA were extracted from infected C6/36 cells using TriSure Isolation Reagent (Roche). The extracted DNA was treated with RNAse and the extracted RNA was treated with DNAse to ensure nucleic acid purity. Using the pan-flaviviral primers, as used for virus screening (Scaramozzino et al., 2001), the DNA sample was tested for the presence of HANKV DNA via direct PCR and the RNA sample was tested via RT-PCR for the presence of HANKV RNA.

To confirm the absence of cell fusion formation and DNA forms in HANKV infected cells, fresh viral cultures were produced (passage 3) in a C6/36 cell line via three dilutions of passage 2 supernatant (*A*=2.10^−1^, *B*=2.10^−2^, *C*=2.10^−3^). Three different pHs (7, 6 and 5), adjusted and buffered with 2 [N-morpholino] ethanesulphonic acid (MES), 10 mM (Flow Laboratories), were tested in culture medium to look for cell fusion as described by Higgs and Gould (1991). Total nucleic acids were extracted using BioRobot EZ1 (Viral RNA Mini kit: Qiagen) and viral replication under each pH condition was confirmed from supernatants and cell pellets by a positive test result (CT 15 -17) in a pan-flaviviral qRT-PCR (Moureau et al., 2007).

The nucleic acid extractions of cells and supernatants (from culture B) were then used to test for HANKV DNA forms. Total nucleic acids from two separate batches of non-infected C6/36 were also used as controls. These nucleic acid extractions were tested in RT-PCR versus direct PCR using seven different primer pairs (Supplementary Table) targeted to HANKV C-prM, NS3 and NS5 genes and to the integrated sequences known to exist in the C6/36 *St. albopicta* genome (Crochu et al., 2004), one pair for regions encoding CSA1 (AF411835), CSA2 (AY223844) and CSA3 (AY223845), respectively.

RT-PCR was performed using an Access RT-PCR kit (Promega), according to the manufacturer's recommendations. The cyclical program of the RT-PCR consisted of 48 °C for 45 min and 94 °C for 2 min, followed by 40 cycles at 94 °C for 30 s, 55 °C for 45 s and 68 °C for 45 s, with a final elongation step at 68 °C for 7 min. The cyclical program of the PCR consisted of 94 °C for 5 min, followed by 40 cycles at 94 °C for 30 s, 55 °C for 1 min and 72 °C for 45 s, with a final elongation step at 72 °C for 7 min. PCR was performed using *Taq* DNA polymerase (Invitrogen).

### Virus sequencing and sequence analysis

Primers targeted to conserved regions of the flavivirus genome were used to amplify partial E (Gaunt and Gould, 2005), NS5 (Chang et al., 1994) and NS3 genes (Grard et al., 2007) of HANKV. Further primers were designed based on the sequences obtained and the available ISF sequences for amplifying and sequencing the remaining parts of the genome (primer sequences available from the authors upon request). RT-PCR products were purified and sequenced directly or cloned when necessary into pGEM-T vector (Promega) for sequencing. The complete genome sequence of HANKV (GenBank accession number JQ268258) was assembled using Sequencher v4.8 (Gene Codes). Phylogenetic analyses were performed for the complete coding sequence and separately for the E, NS5 and NS3 genes including flavivirus sequences available in GenBank. For the region encoding the NS5 gene, a nucleotide “insect-specific focus” data set was prepared that comprised 14 ISFs plus three outgroup viruses, namely tick-borne encephalitis virus (TBEV), Rio Bravo virus (RBV) and DENV (length 918nt, positions 2941–3192 relative to protein reference sequence CFAV NP_041725). For the region encoding the NS3 gene, an amino acid “insect-specific focus” data set was prepared which also comprised 14 “insect-specific” flavivirus taxa and additionally the three outgroup taxa (length 600 aa; positions 1476–2028 relative to reference sequence CFAV NP_041725). Sixty-one sequences were included in the E gene amino acid “insect-specific focus” analysis. No mosquito-borne, tick-borne or NKV sequences were included as outgroups due to high levels of divergence and ambiguous alignment; hence, trees were midpoint rooted. Two E gene region data sets were analysed, with the second undergoing manual trimming at the 3’ end to remove regions of ambiguous alignment (length 431 aa and 385 aa, respectively, positions 279–667 and 279–663 relative to reference sequence CFAV NP_041725). For the analysis of all 76 available flaviviral ORF amino acid sequences plus HANKV, the alignments were subjected to GBlocks stripping to remove regions of ambiguous alignment least stringent (final alignment length 2655 aa) (Talavera and Castresana, 2007).

Additional analyses were performed to include the NS5 gene sequences that recently became available from Spain (Vazquez et al., 2012) (final alignment 780 bp; positions 8934–9014 relative to protein reference sequence CFAV NC_001564 and also the ∼150 bp sequences available from Europe (Calzolari et al., 2012).

All nucleotide and amino acid alignments were conducted using MUSCLE (Edgar, 2004). Phylogenetic analyses were conducted under the Bayesian Markov chain Monte Carlo (MCMC) method implemented in MrBayes v3.1.2 (Huelsenbeck and Ronquist, 2001). For nucleotide data sets, MODELTEST (Posada and Crandall, 1998) was used to select the best-fit model of nucleotide substitution (the GTR+Γ_4_+I model), and for amino acid data sets equivalent amino acid phylogenetic analyses in MrBayes were conducted using the WAG model of amino acid replacement. All parameters were estimated from the data under default priors. Markov chains were run for a minimum of 20 million generations, with the exception of the ORF data set, which was run for 50 million generations and the first 10% of samples were discarded as burnin. Support for nodes was assessed using posterior probability values calculated in MrBayes. All phylogenetic analyses were carried out on the freely available Bioportal server (www.bioportal.uio.no). Stationarity was assessed at effective sample sizes (ESS)>400 using Tracer v1.4.1 (Drummond and Rambaut, 2007).

### Mosquito COI sequence analysis

To attempt identification of the mosquito host species of HANKV, DNA was extracted from the HANKV- positive mosquito pool using DNeasy kit (Qiagen). A section of the mitochondrial COI gene commonly referred to as the “barcode” region was amplified using previously published primers (Folmer et al., 1994) and cloned to pGEM-T vector (Promega). A total of five individual clones was sequenced and found to represent three distinct sequences, namely Contig 1, Contig 2 and Contig 3 (GenBank accession numbers JQ268259-JQ268261). These three COI sequences, originating from mosquitoes from the viral-positive pool, were inserted into a bespoke reference backbone data set of nucleotide sequences that included 530 sequences (636 bp) from across the Culicidae in which each species was represented by, in general, multiple individuals. Sequences were aligned using MUSCLE (Edgar, 2004) and a neighbor-joining tree was generated in PAUP⁎ (Swofford, 2000) (data not shown). A smaller “Aedini-subset” alignment was then prepared. Included were (i) all sequences available for those aedine species recorded from Finland (Utrio, 1979), (ii) all other publicly available aedine species with COI sequences that were relevant as indicated by default searches conducted in BLAST and BOLD resources and (iii) sequences from a number of aedine specimens present in the collections at the Natural History Museum London (Genbank accession numbers JQ246392-JQ246404), (data not shown). Finally, an *Oc. caspius-*focused subset alignment was prepared for clarity. For all subset analyses, the Bayesian Markov chain Monte Carlo (MCMC) method was implemented in MrBayes v3.1.2 (Huelsenbeck and Ronquist, 2001). MODELTEST (Posada and Crandall, 1998) was used to select the best-fit model of nucleotide substitution (the GTR+Γ_4_+I model), all parameters were estimated from the data under default priors and Markov chains were run for a minimum of 20 million generations, with the first 10% of samples discarded as burnin. Support for nodes was assessed using posterior probability values calculated in MrBayes. All phylogenetic analyses were carried out on the freely available Bioportal server (www.bioportal.uio.no). Stationarity was assessed at effective sample sizes (ESS)>400 using Tracer v1.4.1 (Drummond and Rambaut, 2007). The *Oc. caspius-*focused subset results are shown in Fig. 4.

## Figures and Tables

**Fig. NS3 and AAV3:**
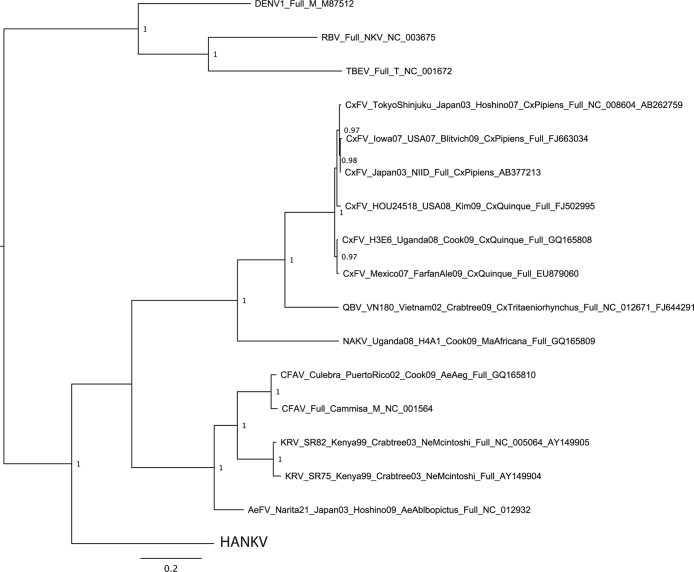
Bayesian phylogeny of the encoded NS3 gene region amino acid data set. Posterior probabilities of ≥0.9 for major nodes only are included and trees are mid-point rooted for clarity. All horizontal branch lengths are drawn to a scale of substitutions per site. HANKV: Hanko virus. The rest of the virus abbreviations are according to ICTV 2011 (Pletnev et al., 2011).
