# Supplementary material for: Novel insect-specific flavivirus isolated from northern Europe
Source: Virology. 2012 Nov 25;433(2):471–8. doi: 10.1016/j.virol.2012.08.038 (PMC3919202; doi:10.1016/j.virol.2012.08.038)
Supplement: Supplementary file 1 — Supplementary data [file mmc2.doc]

| **Primer**  **(S-sense, R-reverse)** | **Genomic position** | **Primer sequence (5’-3’)** | **PCR product length (bp)** |
| --- | --- | --- | --- |
| HANKV_1_S | 361-381 | CCCAGAAAGGTACCAATCATG | 540 |
| HANKV_1_R | 880-900 | CATGGTGTTGTGGTCATGCTT |
| HANKV_2_S | 4927-4946 | GCTGACATAGAAGCTGGAGA | 560 |
| HANKV_2_R | 5467-5486 | TTTCCTTCCGACACATCTCC |
| HANKV_3_S | 7959-7979 | GAACCCAAATGCGGAATTCGT | 530 |
| HANKV_3_R | 8467-8488 | ATGTTGACACATCCGTCATCAT |
| CSA1_A_S | 3124-3143 | CGTCCCGATTGTTCTGATCG | 528 |
| CSA1_A_R | 3651-3632 | GGATGGTAGCCAAAAGTCGT |
| CSA1_B_S | 5945-5964 | GGTGACGTTGTCACCAACGT | 541 |
| CSA1_B_R | 6485-6465 | CTTTTCTGGAGTTGCGAGATT |
| CSA2_C_S | 610-631 | CTGTATATTGGAAACGCCAATA | 386 |
| CSA2_C_R | 995-976 | GTAACAACCTGCCCTGATCC |
| CSA3_D_S | 109-130 | CAGTAAGTATTGTGACTGGACA | 400 |
| CSA3_D_R | 508-489 | CAGCGCTGAGATGATCTTGG |
